# Use of congenital hypertrophy of the retinal pigment epithelium as a clinical sign of familial adenomatous polyposis

**DOI:** 10.5935/0004-2749.2023-0115

**Published:** 2024-10-31

**Authors:** Adriana Amaral Carvalho, Thaísa Soares Crespo, Luciano Sólia Násser, Célia Márcia Fernandes Maia, Cláudia de Alvarenga Diniz Fonseca, Christine Mendes Silveira, Juliana Bastos Amaral, Daniella Reis Barbosa Martelli, Hercílio Martelli Júnior

**Affiliations:** 1 Hospital Universitário Clemente de Faria, Universidade Estadual de Montes Claros, Montes Claros, MG, Brazil; 2 Faculdade de Medicina, Universidade Estadual de Montes Claros, Montes Claros, MG, Brazil; 3 Medicina Oral e Patologia Oral, Faculdade de Odontologia, Universidade Estadual de Montes Claros, Montes Claros, MG, Brazil; 4 Clínica Oftalmológica Luciano Násser, Montes Claros, MG, Brazil

**Keywords:** Retinal diseases/congenital, Retinal pigment epithelium, Hypertrophy/congenital, Adenomatous polyposis coli / genetics, Phenotype, Optical coherence tomography

## Abstract

**Purpose:**

To evaluate the presence of congenital hypertrophy of the retinal pigment epithelium in a large family affected by familial adenomatous polyposis and identify the causative mutation in the adenomatous polyposis coli gene. Thus, we aimed to determine the significance of congenital hypertrophy of the retinal pigment epithelium as a phenotypic marker of the disease.

**Methods:**

A family consisting of 95 individuals was evaluated. Among these, 45 individuals were randomly selected by convenience sampling method to undergo ophthalmological evaluation. A funduscopic exam, including slit lamp and indirect ophthalmoscopy, were performed in the selected patients. In those with retinal lesions, a retinography was obtained. The adenomatous polyposis coli gene was sequenced in one affected family member to identify the pathogenic mutation. Once the variant was identified, six undiagnosed family members were tested for the mutation via capillary electrophoresis sequencing.

**Results:**

Congenital hypertrophy of the retinal pigment epithelium was observed in 13 (28.9%) of the 45 individuals evaluated. Of these, nine patients were confirmed to have familial adenomatous polyposis (via colonoscopy or molecular testing). However, four patients had not been investigated. Of the 32 (71.1%) family members without the lesion, 14 did not have familial adenomatous polyposis and 18 were yet to be evaluated. The lesions were bilaterally present and exhibited a peculiar fish-tail shape in all the evaluated individuals. Adenomatous polyposis coli gene sequencing revealed a pathogenic variant c.4031del. (Ser1344*), in heterozygosity (49.27%), in exon 16.

**Conclusions:**

The study findings confirmed the significance of congenital hypertrophy of the retinal pigment epithelium as a phenotypic marker for familial adenomatous polyposis. Furthermore, it is an effective first-line screening method for at risk family members of such patients. The novel mutation identified in our study participants, which is yet to be described in the literature, causes an aggressive form of the disease.

## INTRODUCTION

Congenital hypertrophy of the retinal pigment epithelium (CHRPE) is a dark pigmented lesion in the retina, which is often surrounded by a halo of depigmentation. Its prevalence in the general population is approximately 1.2-4.4%, and it does not have any clinical significance^(1^-^3)^. These lesions tend to be unilateral in 98% of the patients^(1^,^4)^ and clinically appear as flat and darkly pigmented lesions with well-defined smooth borders. CHRPE can also demonstrate a multifocal presentation, appearing as multiple lesions grouped in a quadrant on fundus examination. This grouping resembles an animal paw or footprint and is therefore commonly referred to as “bear tracks”. Histologically, they appear as a single layer of tall retinal pigment epithelial cells that are filled with large hypertrophic melanosomes^(5^,^6)^.

A differential diagnosis of pigmented lesions in the retinal pigment epithelium is a hereditary disorder called familial adenomatous polyposis (FAP; OMIM# 175100). FAP is a cancer predisposition syndrome that is classically characterized by the development of thousands of adenomatous colorectal polyps in the second decade of life, which can progress to cancer during early adulthood^(3^,^7)^. Although the pigmented ocular fundus lesions in patients with FAP are also referred to as CHRPE, they have different histological and clinical characteristics. Histologically, they are benign hamartomatous malformations of the retinal pigment epithelium. Clinically, the lesions are typically bilateral present, haphazardly distributed, and smaller and more ovoid than CHRPE. Furthermore, they have a jagged edge, with a characteristic area of depigmentation at one edge of the lesion in the shape of a comma or fish-tail^(2^,^4^,^5)^.

To accurately differentiate pigmented fundus lesions in FAP individuals from the original CHRPE in individuals without FAP, several authors have proposed alternative terms. Hennessy et al. proposed to call these lesions “multiple retinal pigmented epithelial hamartomas” when CHRPE was observed in individuals with FAP^(8)^. However, Traboulsi et al. referred to these lesions as “pigmented lesions of the ocular fundus”^(9)^. Shields et al. proposed calling the lesions in patients with FAP as “retinal pigment epithelium hamartomas associated with familial adenomatous polyposis”^(10)^. Bonnet et al. recently proposed using the term FAP-associated CHRPE because it more accurately correlates with the genetics, histopathology, and clinical presentation of the lesions^(6)^. However, these lesions in patients with FAP have still been referred to as CHRPE in the scientific literature^(10^-^12)^.

Classic FAP is an autosomal dominant condition with 100% penetrance. Its pathogenesis is linked to germline mutations in the adenomatous polyposis coli (*APC*) gene located on chromosome 5q21-22^(13)^. Attenuated FAP (AFAP; OMIM# 175100) is a milder phenotypic variant of FAP in which patients have fewer polyps (<100) on colonoscopy^(13)^. Patients with FAP also exhibit several extracolonic manifestations such as polyps in the upper digestive tract (stomach and duodenum), small intestine, thyroid, adrenals, pancreas, and pituitary gland, epidermal inclusion cysts, sebaceous cysts, lipomas, dental abnormalities, retinal pigmentary lesions, tumors of the endocrine system, central nervous system, and liver, adenomas and adenocarcinoma of the biliary tree and duodenal papilla, desmoid tumors, and osteomas^(12)^.

The aim of this study was to identify CHRPE lesions in patients diagnosed with FAP and their relatives who belonged to a large Brazilian family. Additionally, we aimed to describe the pattern of distribution and characteristics of the lesions, which will help in clarifying the role of CHRPE as a phenotypical marker of FAP. Furthermore, we identified the germline mutation in the *APC* gene that caused the disease.

## METHODS

**Figure 1:**
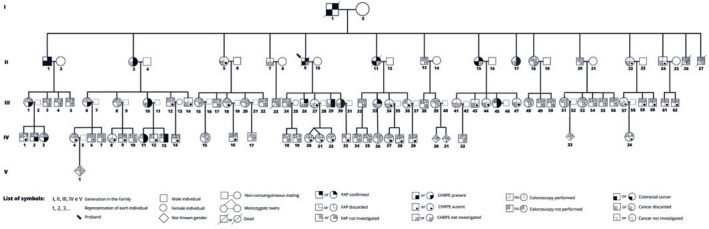
Pedigree of the family with familial adenomatous polyposis. Five generations of a family, consisting of 95 members, from North Minas Gerais, Brazil, were recruited for the present study. The black arrow denotes the proband. Forty-five patients were evaluated for congenital hypertrophy of the retinal pigment epithelium, and it was found in 13 of them. The square (male) and circle (female) symbols were divided into 4 quadrants, each representing a different situation: familial adenomatous polyposis carrier (yes, no, or not investigated), congenital hypertrophy of the retinal pigment epithelium (present, absent, or not investigated); colorectal cancer (present, absent, or not investigated), and colonoscopy (performed or not). Symbols with diagonal lines represent deceased individuals.

This study was a cross-sectional and descriptive study. The proband with FAP was referred by a gastrointestinal surgeon from the research group. Five generations of the patient’s family (comprising of 95 members) from the northern region of Minas Gerais State, Brazil, were analyzed. Details of the family health history were collected, and a pedigree chart was constructed (Figure 1). The father had died of colon cancer at the age of 55 years and was probably the carrier of the affected gene. He may have been unaware that he was a carrier of the genetic condition. The index case belonged to the second generation of the family. He was diagnosed with FAP after colorectal cancer (CRC) was detected, and it was associated with several colonic polyps. The study was carried out from December 2018 to April 2022.

**Figure 2:**
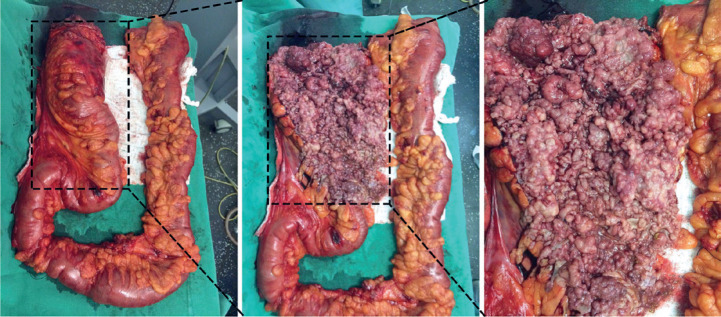
Surgical specimen obtained after a total proctocolectomy in a patient (patient III-10 in the family pedigree) with familial adenomatous polyposis. In the figure, thousands of polyps were observed in the colon.

During the study, colonoscopy was performed in 12 individuals who belonged to the family. Family members aged <12 years were excluded from the examination according to the restriction imposed by the health establishment where the aforementioned exams were performed. Other family members had already undergone a colonoscopy at the beginning of the study. FAP was diagnosed on the basis of the identification of >100 colonic polyps on colonoscopy (Figure 2) and a histopathological report confirming adenomatous polyps.

All family members were invited to undergo an ophthalmological evaluation regardless of a confirmed diagnosis of FAP or age. A total of 45 individuals were examined by an ophthalmologist for CHRPE via a funduscopic examination, including slit lamp and indirect ophthalmoscopy (with 90 and 20 diopter lenses, respectively), after the pupil was dilated with 1% tropicamide. The visual acuity of all the individuals was also evaluated. The lesion characteristics such as number, location in the retina, shape, and laterality were evaluated and documented in the medical records by the ophthalmologist, who was unaware of the patient’s disease status. In individuals with retinal lesions, fundus photographs were obtained (Retinographic Canon^®^; *Canon* Inc., Ōta, Tokyo, *Japan*). All retinography images were analyzed by the same ophthalmologist and the characteristics of the lesions were documented.

On the day of ophthalmologic evaluation, the patients also underwent a clinical examination by a general practitioner and an oral cavity examination by a dentist. These were performed to identify any extracolonic manifestations of FAP other than CHRPE.

Genetic analysis was performed to identify the mutation associated with FAP in this family. The *APC* gene was sequenced, and the genomic DNA was isolated from oral epithelial cells. After the genomic DNA was extracted and fragmented, it was indexed and captured with custom probes. Furthermore, the regions of interest were enriched. After next generation sequencing of the target sequences (Illumina), they were aligned and variants were detected on the basis of the GRCh37 version of the human genome. The generated data were analyzed using customized bioinformatics processes (germline pipeline version 3.6). The variants were interpreted by considering the patient’s clinical condition and the variant classification protocol of the American College of Medical Genetics.

Once the pathogenic mutation was recognized, DNA sequencing was performed via capillary electrophoresis in the region around the identified variant. This was performed to identify the mutation in six family members who had not undergone a colonoscopy but had one parent who was affected by the disease.

The study was approved by Institutional Review Board (No: 97635318.6.0000.5146 in September 2018).

## RESULTS

Among the 45 (47.4%) family members who underwent an ophthalmological evaluation, CHRPE was observed in 13 members (28.9%). Of the 13 members, 9 (69.2%) had a confirmed diagnosis of FAP (by colonoscopy or molecular test) and 4 (30.8%) had not yet been evaluated. Of the other 32 members (71.1%) without CHRPE lesions, 14 (43.8%) did not have FAP (normal colonoscopy or negative molecular test) and 18 (56.2%) had not undergone colonoscopy or molecular testing. Of these 18 members, 12 were children.

**Figure 3:**
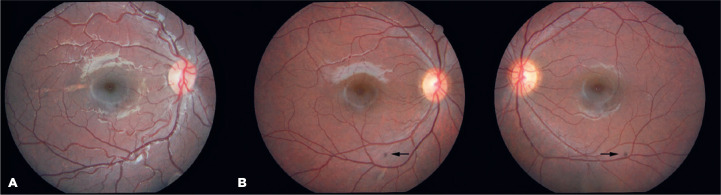
Fundus images of study participants that were obtained via a digital retinography system. A) Normal retina. B) Punctate congenital hypertrophy of the retinal pigment epithelium (CHRPE) in both eyes (dot lesions) (patient IV-2 in the family pedigree).

**Figure 4:**
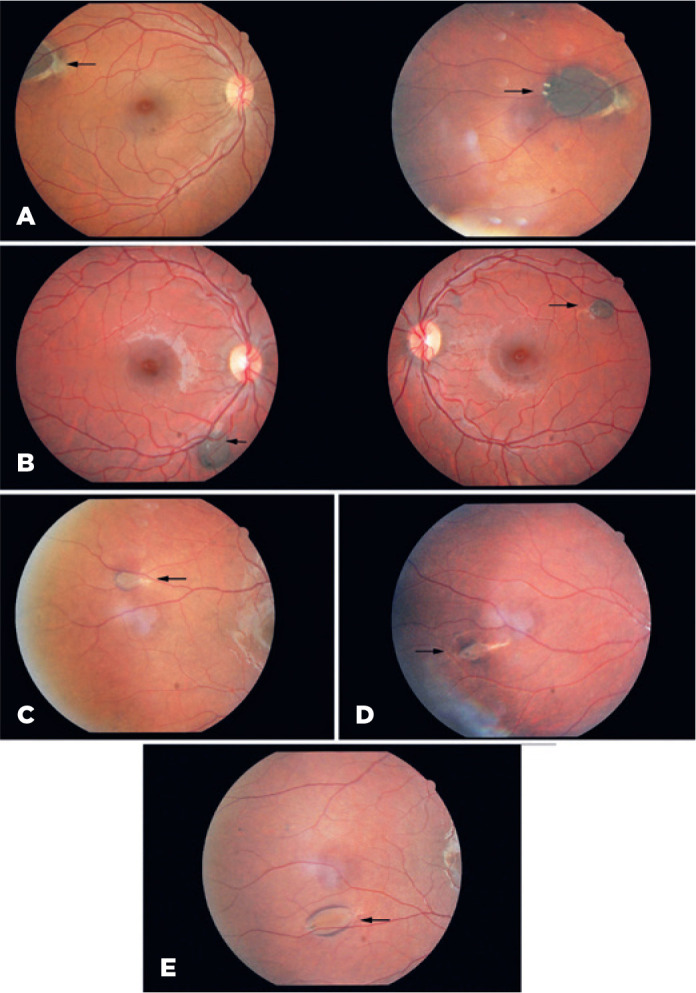
Retinal fundus images of study participants that were obtained via digital retinography. A, B) Congenital hypertrophy of the retinal pigment epithelium appears hyperpigmented, with fish-tail depigmentation at one margin of the lesion (patients II-17 and IV-13, respectively in the family pedigree). C, D) Congenital hypertrophy of the retinal pigment epithelium appears hypopigmented, with fish-tail depigmentation at one margin of the lesion (patients III-1 and IV-2, respectively in the family pedigree). E) congenital hypertrophy of the retinal pigment epithelium appears hypopigmented, with fish-tail depigmentation at one margin of the lesion and a hyperpigmented halo (patient IV-13 in the family pedigree).

**Figure 5:**
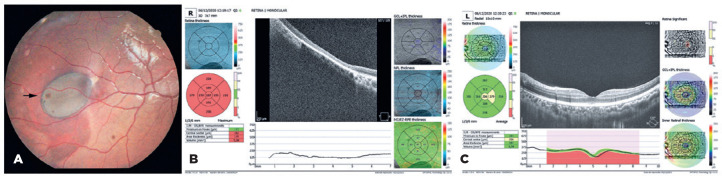
Fundus images of a study participant (patient III-30 in the family pedigree) obtained through digital retinography. A) Large hyperpigmented lesion at the periphery of the retina needed to be differentiated from melanoma. B, C) Computed tomography of the same lesion.

CHRPE lesions were observed bilaterally in all the patients. The number of lesions in the right eye ranged from 1 to 4 and that in the left eye ranged from 1 to 3. The total number of lesions in both eyes ranged from 2 to 6 regardless of age. The lesions varied in size from punctate (dot) lesions (Figure 3) to lesions measuring 0.25-4 disk diameters. Except for the dot lesions, all other lesions were oval in shape. Fish-tail depigmentation at one margin of the lesion was observed in all the patients (Figure 4). Variation in the pigmentation within the lesions was also noted. In one patient, computed tomography (CT) was performed to differentiate the CHRPE-type lesion from a melanoma (Figure 5).

The extracolonic manifestations detected in the study were osteoma (n=4), isolated café-au-lait spots (n=8), and epidermal cysts (n=2). Ophthalmoscopy revealed papilledema in one patient. Magnetic resonance imaging (MRI) was immediately obtained in this patient, which revealed hydrocephalus. Thus, an endoscopic ventriculostomy was performed. No tumor-associated lesions were observed on the MRI or during the surgical procedure, excluding the possibility of a brain tumor-associated with FAP (Turcot syndrome)^(11)^. Colonoscopy performed eleven months after the ophthalmological evaluation did not demonstrate colonic “polyposis”.

Genetic tests in one of the affected family members (Patient III-20, Figure 2) revealed a mutation that has not yet been described in the literature. Gene sequencing revealed a pathogenic variant c.4031del. (Ser1344*), in heterozygosity (49.27%), in exon 16 of the *APC* gene. This variant may have changed the reading frame (frameshift) by promoting the substitution of an amino acid, which resulted in the early arrest of protein translation and formation of a truncated protein. The ophthalmological evaluation in this patient revealed CHRPE. Because of the peculiarity of the retinal lesion, a CT was performed to differentiate the CHRPE-type lesion from a melanoma.

Genetic testing in the six family members who did not undergo colonoscopy revealed the presence of the variant in only three of them. The ophthalmological examination of these six patients revealed CHRPE lesions only in the three individuals in whom the mutation was detected.

## DISCUSSION

FAP accounts for only approximately 1% of all the cases of CRC. Nonetheless, the lifetime risk of CRC in patients with untreated FAP is almost 100%^(6^,^13)^. CRC in patients with FAP develops at a mean age of 44 years^(14)^. Premature death in an *APC* mutation carrier can be prevented by performing prophylactic proctocolectomy before a malignancy develops. However, the symptoms of FAP may only appear in advanced stages^(15)^. Furthermore, these may be non-specific^(15^,^16)^. Thus, individuals who do not have an index case in the family that triggers the screening of relatives will remain undiagnosed until the disease has progressed to an advanced stage^(16)^. Therefore, these patients have a poor prognosis.

The family evaluated in this study had a clinical course consistent with this description. The nine patients who were diagnosed via a colonoscopy that was performed during middle adulthood had no gastrointestinal symptoms until one or two years before their diagnosis. The onset of symptoms coincided with the identification of extensive polypoid disease on colonoscopy or the detection of cancer. Furthermore, intestinal bleeding was the main symptom in these nine patients. Three teenage siblings who underwent colonoscopy were diagnosed with extensive polypoid disease at an early age (12, 14, and 17 years). Despite the diffuse polyposis on colonoscopy, they had no significant intestinal symptoms. However, the oldest teenage sibling reported occasional abdominal pain and episodes of diarrhea after eating. In view of the extensive colorectal polyposis in colonoscopy, the three adolescents required a colectomy. The procedure was performed shortly after the colonoscopy on the three brothers, aged 16, 14 and 18 respectvely.

Because early diagnosis is essential for a satisfactory clinical evolution of patients with FAP, the evaluation of potential early and non-invasive phenotypic markers of the disease is of great clinical importance. Screening patients before the onset of clinically detectable polyposis ensures the best prognosis in patients. Furthermore, it helps to persuade individuals at risk to regularly follow-up with the physicians and improve their adherence^(2^,^6)^. Among the extracolonic manifestations of FAP, CHRPE is the phenotypic marker commonly used for screening. It is evaluated via an ophthalmologic examination that is easy to perform, safe (non-invasive), relatively low in cost, well accepted by patients, and less stigmatized than colonoscopy^(2)^. Furthermore, CHRPE is the first extracolonic manifestation of FAP^(6)^. In our study, retinal change was identified in a one-month-old child. CHRPE had develop at birth or shortly thereafter in up to 80% of patients^(11).^

CHRPE may not be observed in all the patients with FAP because its manifestation is linked to mutations in some specific codons of the *APC* gene^(11)^. Thus, the absence of CHRPE in an individual at risk does not exempt him from further investigation via colonoscopy or genetic tests^(6)^. Nonetheless, the global prevalence of CHRPE in individuals with the *APC* gene mutation is 90%^(11^,^17)^.

In families with FAP and CHRPE, identification of these lesions in their relatives is an applicable screening method. Numerous authors have stated that CHRPE is a reliable indicator of FAP carrier status^(6)^. However, intrafamilial variability, which may be attributable to incomplete penetrance of the CHRPE phenotype, has been reported in some families^(11)^.

In a systemic review, Bonnet et al. (2022) determined that the sensitivity (mean 79%) and specificity (mean 89%) of FAP-associated CHRPE is highly variable. Thus, as a marker, CHRPE is less likely to produce false-positive results. However, it may be prone to false-negatives^(6)^. Baba et al. postulated that the differing reliability values may be related to the methods used for ophthalmological evaluation, as well as sampling errors due to the small sample sizes^(6^,^18)^. Furthermore, when considering certain characteristics of the ocular lesions, especially “high-risk” features for FAP (such as bilaterality and multiple lesions), the relative specificity of CHRPE as a marker increases^(6)^. In our study, 23 of the 45 (51.1%) individuals who underwent ophthalmological evaluation had their diagnosis of FAP confirmed (n=9, 42.9%) or excluded (n=14, 57.1%) by colonoscopy or molecular analysis. Consistent with the research hypothesis, CHRPE lesions were detected in the nine patients with a confirmed disease. Likewise, the lesions were not detected in the 14 patients in whom FAP was excluded.

In our study, the CHRPE lesions were observed bilaterally^(6)^ and with fish-tail depigmentation at one of the margins^(2)^ in patients with FAP. These characteristics are not observed in the CHRPE lesions that occur sporadically in the general population. Thus, this finding is clinically significant for the diagnostic suspicion of FAP, as evidenced in this study. Furthermore, the presence of multiple lesions (cumulative total of ≥3 in one or both eyes)^(19)^, and the size of the lesion^(11)^, with at least one lesion >0.5 in disc diameter^(19)^, are also significant lesion characteristics in FAP that were observed in all our study participants.

Another characteristic pattern of FAP-associated CHRPE is its location in the retina. Bonnet et al. determined that 84% of the lesions are located in the retinal periphery; the rest (16%) are located within the posterior pole^(6)^. Similarly, all the CHRPE lesions in our study were located in the mid-peripheral or far peripheral retina. No lesions were detected in the posterior pole. These findings indicate that CHRPE may not be detected if only a standard ophthalmologic evaluation is performed without obtaining a wide-field image of the retina^(20^,^21)^.

Regarding extra-colonic manifestations, three of our study participants reported having undergone an osteoma removal previously. In two patients, the lesion was on the face, and in the third patient, the lesion was on the hard palate. None of these patients were counseled about the possibility of an associated systemic disease. Thus, although the progress in performing genetic tests in these patients has been significant, it is crucial to disseminate scientific knowledge among health professionals. This will ensure that patients are diagnosed in a timely diagnosis, and preventive treatment is administered.

Another applicability of identifying CHRPE in patients with FAP is to signal the location of the mutation in the APC gene, and thus direct the genetic tests to be performed. The presence of CHRPE has been associated with mutations between codons 463 and 1393^(6^-^11)^. The presence of osteomas (observed in some of our patients) has been associated with mutations in codons 767 to 1578^(22)^. Therefore, the mutation associated with FAP in this family should have been between codons 767 to 1393. This knowledge is valuable at the time of molecular analysis because it allows us to request a more specific genetic study, increasing the testing efficiency and lowering the testing costs. Furthermore, knowing the exact location of the mutation may aid in predicting the probable clinical evolution and monitoring the patient. In addition, it may indirectly provide information regarding the severity of the disease, age at onset, and extent of the disease. Sequencing of the *APC* gene in one family member revealed a pathogenic variant c.4031del. (Ser1344*). This location is consistent with the expected location that was determined on the basis of the clinical manifestations of the disease in this family. The variant identified in this family was not found in the dbSNP, population control bank (gnomAD)^®^, ClinVar, or previous studies. In silico predictors indicate that the effect of this variant is undetermined.

The study findings confirm the significance of CHRPE lesions as a possible phenotypic marker for patients with FAP and as an effective screening method for their at risk family members. Bilaterally present, multiple, oval lesions with fish-tail depigmentation at one margin increases the specificity of CHRPE as a marker for FAP, which can guide the ophthalmologist in making a diagnosis. Because these lesions are predominantly located in the peripheral retina, it is crucial to perform retinal mapping in all the patients at risk for FAP or other systemic diseases. This will allow early diagnosis and benefit the patient. A novel pathogenic germline variant c.4031del:p(Ser1344*) was identified in exon 16 of the *APC* gene in our study. This mutation, which has not yet been described, is associated with profuse FAP that manifests at an early age.
